# Enhancement of EPDM Crosslinked Elastic Properties by Association of Both Covalent and Ionic Networks

**DOI:** 10.3390/polym13183161

**Published:** 2021-09-18

**Authors:** Chloé Larrue, Véronique Bounor-Legaré, Philippe Cassagnau

**Affiliations:** Ingénierie des Matériaux Polymères, Univ-Lyon, Université Claude Bernard Lyon 1, 69622 Villeurbanne, France; chloe.larrue@etu.univ-lyon1.fr (C.L.); veronique.bounor-legare@univ-lyon1.fr (V.B.-L.)

**Keywords:** crosslinked EPDM, ionic network, compression set

## Abstract

The objective of this study was to replace elastomer crosslinking based on chemical covalent bonds by reversible systems under processing. One way is based on ionic bonds creation, which allows a physical crosslinking while keeping the process reversibility. However, due to the weak elasticity recovery of such a physical network after a long period of compression, the combination of both physical and chemical networks was studied. In that frame, an ethylene-propylene-diene terpolymer grafted with maleic anhydride (EPDM-g-MA) was crosslinked with metal salts and/or dicumyl peroxide (DCP). Thus, the influence of these two types of crosslinking networks and their combination were studied in detail in terms of compression set. The second part of this work was focused on the influence of different metallic salts (KOH, ZnAc_2_) and the sensitivity to the water of the physical crosslinking network. Finally, the combination of ionic and covalent network allowed combining the processability and better mechanical properties in terms of recovery elasticity. KAc proved to be the best ionic candidate to avoid water degradation of the ionic network and then to preserve the elasticity recovery properties under aging.

## 1. Introduction

The challenge of obtaining elastomers’ reversible crosslinking to meet classical thermoplastics melt processing is elegant and of importance in terms of industrial applications and circular economy. This concept of reversibility is most of the time associated with the possibility of processing at high temperatures (160–250 °C) while maintaining elastic properties at moderate temperatures (−30 to 120 °C). These temperature ranges are indicative and depend on the systems and applications, but for the automotive market, significant elastic properties are currently required up to temperatures of 120 °C (ASTMD 395 standards, for example).

In terms of elastic recovery and processability at high temperatures, Thermoplastic Elastomers (TPE) meet these requirements on the principle of reversible networks based on a chemical nature and structuration. Two types of TPE can be defined. First, some block copolymers present soft blocks with elastomeric behavior and rigid blocks that act like a thermoplastic phase. This specific composition and nature will lead to phase separation, which causes remarkable elastic properties at room temperature. The best known of these TPE is poly(styrene-butadiene-styrene block) block copolymer (SBS). It is composed of polybutadiene blocks (*T*_g_ ≈ −100 °C) and polystyrene blocks (*T*_g_ ≈ +100 °C). Below the glass transition of the polystyrene (PS), this material behaves as a crosslinked rubber [1] due to the widely separated phases at room temperature. Beyond the *T*_g_ of the PS phase, the material can be a priori processed. However, this also leads to a loss in the elastic recovery properties [2,3]. Actually, thermoplastic elastomers have weak elastic properties at temperatures above 100 °C and specifically under long times of compression.

Secondly, a polymer blend with a thermoplastic and an elastomer permit combining the properties of each component. Indeed, the idea is to associate a crosslinked elastomer phase to a thermoplastic phase in order to give the final material both elasticity and processing properties. This objective has been achieved using the dynamic crosslinking process [4], which consists of crosslinking a major elastomer phase concomitantly to its blending with a minor thermoplastic phase. Finally, the crosslinked elastomer is dispersed in the thermoplastic phase. This has led to the emergence of Thermoplastic Vulcanizated (TPV), which has been a real industrial success. Several TPV materials have been developed in recent years, but the most widespread are still based on reactive blends from polypropylene (PP) and ethylene-propylene-diene terpolymer (EPDM) [5,6,7]. Despite their success, these TPVs have some limitations due to sub-products of reactions such as organic volatile compounds.

Another possibility could be to turn to reversible chemistries. The reversible reactions of Diels–Alder [8,9] have often been considered, and recently, the concept of “vitrimers” [10,11,12] has opened up new fields of research and potential applications. However, these chemistries are still not mature for industrial applications because of their cost and the steps they require (chemical modification, grafting, etc.).

Ionomer-based polymer materials were developed several years ago from the pioneering works of Eisenberg [13,14]. These polymers contain a small number of ionic groups (up to 15%mol) pendant or incorporated in the backbone [15]. The ionic groups (in most studies based on maleic anhydride [16,17,18] or other carboxylate groups [19,20,21]) tend to form ionic aggregates, which act as physical crosslinks. The reversibility is related to the increased mobility of ion pairs within ionic clusters under high temperature and shear stress allowing melt processing. At the same time, elastic properties at lower temperature are expected by recovering the cluster morphology by phase separation. Several studies have shown that the neutralization of these ionomers with different salts can influence the mechanical properties. For example, Van der Mee et al. [17] showed the improvement of the compression set at room temperature on EPDM-g-MA (88% without salts versus 15% for a K ionomer and 22% for a Zn ionomer both neutralized at 100%). Choi et al. [22] also worked on EPDM-g-MA neutralized with ZnO. They showed the increase in the crosslink density with the quantity of zinc. The compression set at room temperature is also decreased at 72% with 5 phr of ZnO. Rousseaux et al. [23] showed the influence of the concentration in salt on polypropylene grafted with maleic anhydride (PP-g-MA). As a result, the shear elastic modulus of the ionomers neutralized with NaOH increases from 1 × 10^3^ Pa for a neutralization degree (ND) of 10% to 2 × 10^5^ for a ND of 70%. Spencer et al. [20] showed similar results from ethylene methacrylic acid copolymers. The tensile modulus increases from 0.05 GPa without neutralization to 0.45 GPa for the K ionomers and to 0.40 GPa for the Na ionomers (both neutralized at 40%). In both cases, a plateau is observed at this neutralization degree. This type of technology was also used to develop materials with good shape memory capability such as those of Salaeh et al. [24] or Wang et al. [25].

Unfortunately, elastic recovery properties are generally required at high temperatures, typically in the temperature range 80–120 °C. Under these test conditions, the use of the elastomers-based ionomer is not suitable or at least very restricted. This has already been pointed out by Mora-Barrantes et al. [21]. Actually, to meet this challenge, they then proposed the principle of a combined covalent crosslinking network, i.e., a radical formed network combined with an ionic network. They used a carboxylated nitrile rubber (XNBR) with magnesium oxide (MgO), which will neutralize the carboxylic groups and form the ionic network. They demonstrated that the appropriate combination of covalent and ionic crosslinking allowed controlling the structure of the network of ionic elastomers in order to obtain suitable properties for these materials. They managed to improve the tensile properties of the samples in comparison of the covalent systems. For example, in their system, the modulus at 300% deformations increases from 2.3 MPa with a covalent system to 12 MPa combining both ionic and covalent systems. Unfortunately, the properties are studied until 70 °C and do not answer to the problematic at very high temperature.

Based on this same concept, the objective of the present work is to study the elastic recovery properties (compression set at *T* = 100 °C) and the processability of an EPDM-g-MA crosslinked by a twin network, i.e., a covalent network coupled with an ionomer network. We will study successively the covalent network and the ionic network before scrutinizing their combination and its impact on the expected physical properties. Different metallic salts will be studied as well as their sensitivity to hot water (*T* = 80 °C) from the dependence the equilibrium modulus, the gel fraction, and the compression set.

## 2. Experimental

### 2.1. Materials

The copolymer used is an EPDM grafted with maleic anhydride (EPDM-g-MA, Royaltuf 498) supplied by Addivant. The characteristics of this copolymer are listed in Table 1.

Dicumyl peroxide (DCP) (purity 98%, provided by Sigma-Aldrich), potassium hydroxide (KOH) (purity 90%, provided by Sigma-Aldrich), zinc acetate (ZnAc_2_) (purity 95%, provided by Carl Roth), potassium acetate (KAc) (purity 99%, provided by Fisher Scientific), sodium acetate (NaAc) (purity 99%, provided Sigma-Aldrich), and toluene (purity 99.5%, provided by Carlo Erba) were used as received.

### 2.2. Samples Preparation

The samples were prepared by melt blending in a Haake Polylab internal mixer (Haake Rheomix). The mixing speed was set to 50 rpm at 100 °C. The EPDM-g-MA was first added. Then, after 5 min of mixing, the metallic salt is added and mixed for 5 min. Finally, the DCP is added, and the blend is mixed for 3 additional minutes. The compositions of the different formulations are summarized in Table 2. The protocol, when only one crosslinking agent is used, is similar to the previous one: addition of the metallic salt after 5 min or of the DCP after 10 min of mixing.

The neutralization degree (ND) corresponds to the theoretical degree of neutralization that is targeted for the blend. Thanks to this value, we can calculate the mass of metallic salt that we have to introduce in the formulation following Equation (1):

(1) $$m_{M^{+}}=\frac{0.01\times m_{pol}\times \alpha \times \mathrm{ND}}{M_{MA}\times 100}$$

Here, $\alpha =\frac{q_{MA}}{q_{M^{+}}}$, and *q* is the electric load of the species.

The samples were prepared by compression molding at 180 °C, using a press Polysat 200T. A pre-heating is realized for 3 min, during which the pressure is slowly increased by 50 bars increment from 50 until 200 bars. The samples are cured for 30 min in order to perform the covalent crosslinking from peroxide decomposition. Note the half-time decomposition of DCP at 180 °C is 47 s, based on Msakni et al. [26] data.

### 2.3. Characterization Methods

Processability: The ability of the crosslinked formulation to be processed in subsequent steps was tested by elaborating a film under 200 bars at 180 °C. This test is quite qualitative, but it allowed mimicking a processing operation. Qualitatively, if a thin (≈200 μm) homogenous film is obtained without any aspect surface defaults, it will then conclude that this formulation is suitable for melt processing. On the contrary, if a heterogeneous aspect of the film is observed under these conditions, it can be admitted that this material could not be process in an extruder, for example.

Rheological properties: The complex shear modulus ($G^{*}\left(\omega \right)=G^{\prime }\left(\omega \right)+jG^{\prime \prime }\left(\omega \right),\;\;j^{2}=-1$) was measured using a Discovery Hybrid Rheometer (DHR) from TA instruments. Frequency sweep tests were performed at 160 °C under a strain rate of 4% (linear regime) over an angular frequency ranging from 100 to 0.01 rad/s. The analysis is performed under nitrogen atmosphere using a parallel plate geometry (Ø = 25 mm).

The value of the equilibrium modulus *Ge* was determined by extrapolation from the low frequency plateau exhibited on *G*′ plots ($Ge=\underset{\omega \to 0}{\lim}G^{\prime }\left(\omega \right)$). However, for the weakest crosslinked networks, it is not possible to extrapolate the modulus at equilibrium in a suitable way. In these cases, the value of the storage modulus *G*′ measured at the lowest frequency, i.e., *ω* = 0.01 rad/s will be reported. 

Compression set: The compression set (CS) was measured to determine the capacity of the sample to recover its initial shape. It was measured on cylindrical samples with a diameter of 13 mm and a thickness of approximately 6 mm. The samples are submitted to a strain of 25% at 100 °C for 24 h. The CS was determined after a relaxation time of 30 min at room temperature, according to the ISO 815-1 standard. It is calculated using the following equation:

(2) $$\mathrm{CS}\;\left(\%\right)=\frac{1}{\varepsilon_{0}}\;\left(1-\frac{l_{1}}{l_{0}}\right)\times 100$$

where *ε*_0_ is the deformation imposed (*ε*_0_ = 0.25), *l*_0_ is the sample thickness before the experiment, and *l*_1_ is the sample thickness after 24 h of compression and 30 min cooling at room temperature.

Gel Fraction: The gel fraction (GF) is determined by immersing samples between 60 and 80 mg (*w*_0_) in toluene at 80 °C for 48 h. The samples are weighted after a drying period of 24 h at 40 °C under vacuum (*w_d_*). It is calculated by the following equation:

(3) $$\mathrm{GF}=\frac{w_{d}}{w_{0}}$$

Water aging: In order to determine the water aging of the samples, the different samples (cylindrical samples with a diameter of 13 mm and a thickness of approximately 6 mm) have been immersed in hot water for 48 h at 80 °C. Then, the mechanicals tests and gel fraction determination are realized in subsequent experiments.

## 3. Results and Discussion

### 3.1. Covalent Crosslinking

**Figure 1:**
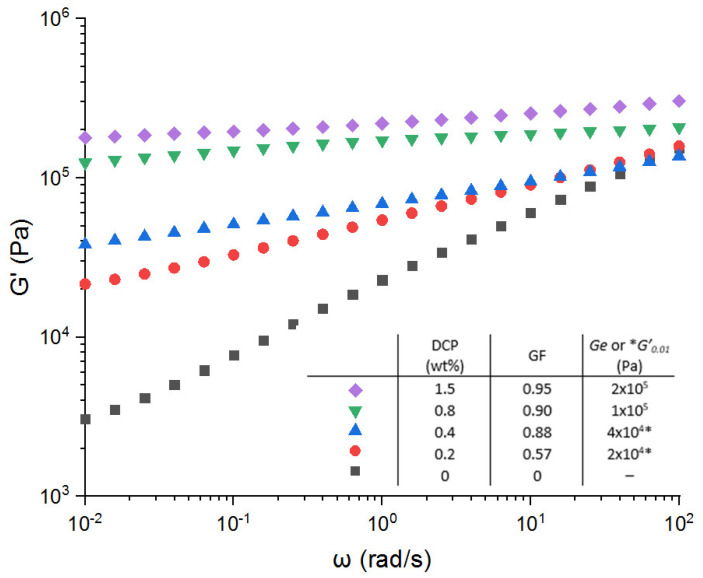
Variation of the storage modulus versus angular frequency for the EPDM-g-MA crosslinked with different peroxide concentration (wt.%). No metallic salt has been added in these formulations.* is *G*′_0_._01_ reported instead of *Ge*.

Figure 1 shows the dependence of the storage modulus on the peroxide concentration used to crosslink the EPDM-g-MA. As expected, it can be observed that the higher the peroxide concentration, the higher the storage modulus. This is consistent with the results of Le Hel et al. [27]. Actually, the regime of the permanent elasticity is not reached at the lowest frequencies, or in other words, a dissipative phenomenon still exists due to the low crosslinking degree and/or the imperfections of the chemical network.

Furthermore, from a processability aspect, the samples can be transformed into a uniform film for the lowest DCP concentrations, 0.2 and 0.4 wt.%, respectively. For the highest DCP concentrations (0.8 and 1.5 wt.%), it was not possible to reach a homogeneous film anymore. Consequently, the concentration of 0.4 wt.%, which corresponds to a gel fraction of 0.88, appears to be the critical one in view of the processability of the crosslinked EPDM samples.

From a quantitative point of view, the maximum crosslinking density obtained for a concentration of 1.5% DCP was calculated according to $Ge=\nu_{R}\cdot R\cdot T\cdot GF^{1/3}$, where *Ge* is the equilibrium modulus, *ν_R_* is the crosslinking density, *R* is the gas constant, *T* is the absolute temperature, and GF is the gel fraction.

From this equation, we obtain a crosslinking density of 50 mol/m^3^ for 1.5 wt.% of DCP, which is in agreement with previous work on the crosslinking of EPDM [27]. For 0.2 wt.% DCP, the crosslinking density is less than 7 mol/m^3^. This calculation was done using *G*′ at ω = 0.01 rad/s, so the crosslinking density is overestimated.

**Figure 2:**
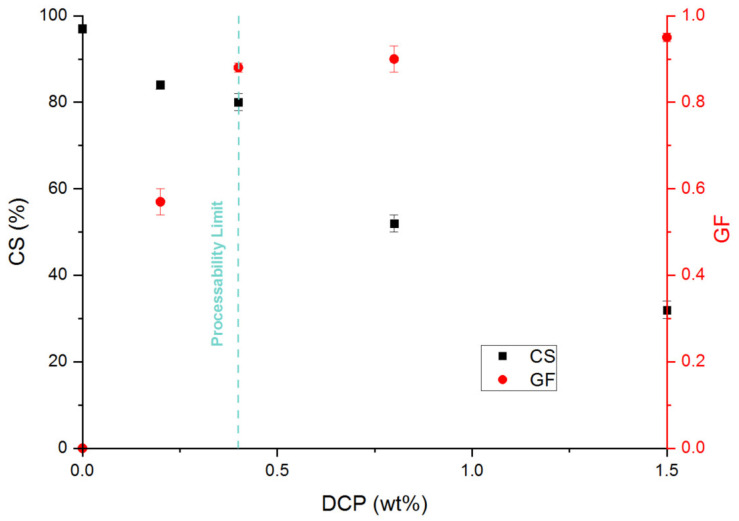
Variation of the compression set (■) and the gel fraction (●) for the EPDM-g-MA crosslinked with different DCP concentration (wt.%). No metallic salt has been added in these formulations.

The compression set aims to quantify the elasticity recovery at long times under a constant deformation. Actually, a compression set not equal to zero proves a dissipative phenomenon at long times. This dissipative behavior depends on different parameters such as the crosslinking density and the nature of the crosslinking chemistry used. It has been demonstrated that the higher the crosslinking density, the better the compression set [27,28]. In terms of chemistry, the radical chemistry leads to the formation of an imperfect network with the presence of dangling chain ends that are at the origin of this dissipative behavior [28,29]. Consequently, and as expected, the compression set (Figure 2) decreases with increasing the gel fraction and so the crosslinking density. The dangling chain ends becomes shorter. The recovery elasticity is more pronounced with increasing the crosslinking density, which is proportional to the DCP concentration [27]. 

In the case of a radical crosslinking, we can go down to a compression set around 30%, but to the detriment of the sample processability. Finally, these results mean that a processable sample crosslinked with DCP cannot have a compression set lower than 80%.

### 3.2. Ionic Crosslinking

**Figure 3:**
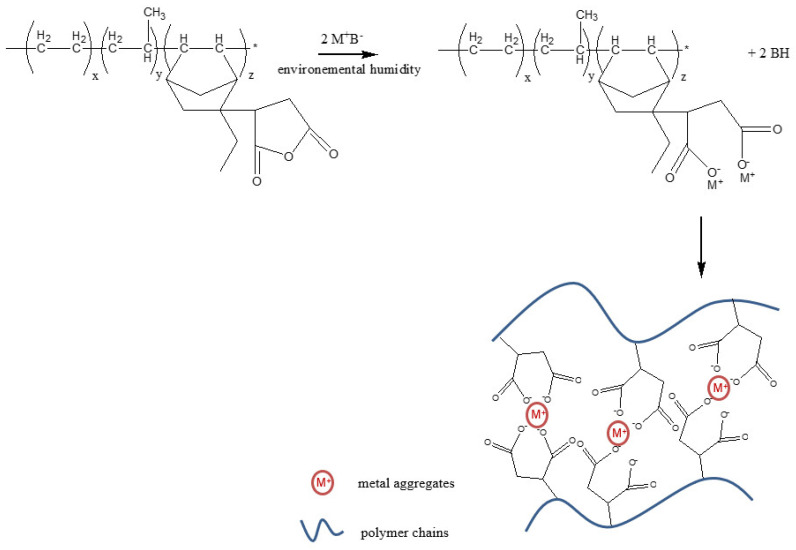
EPDM-g-MA neutralization step, according to Li et al. [30].

First, we studied the potentiality of KOH to react with the grafted maleic anhydride (EPDM-g-MA) according the reaction scheme in Figure 3. Note that the sample with a neutralization degree of 100% in KOH was transformed in the form of powder inside the mixer chamber. This means that the crosslinking is too important and the sample is not processable anymore.

**Figure 4:**
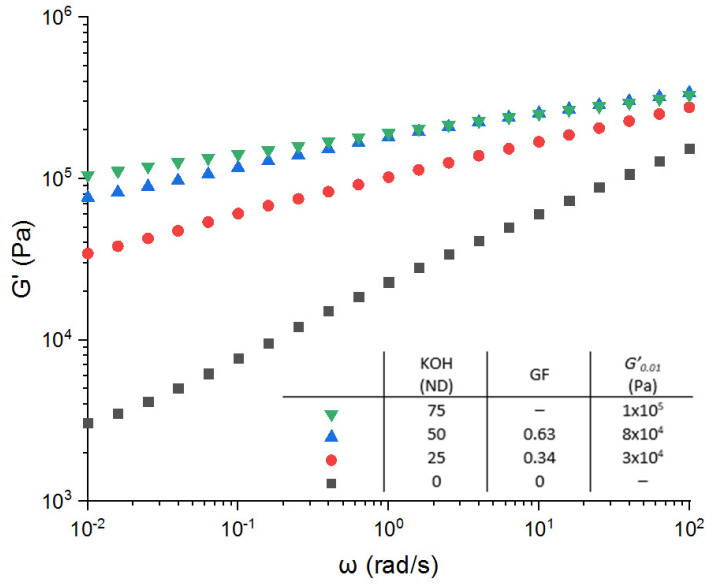
Variation of the storage modulus versus angular frequency for the EPDM-g-MA crosslinked with different neutralization degree of KOH. No DCP has been added in these formulations.

Based on these first results, the impact of the KOH concentration (with a maximum ND of 75%) on the evolution of the storage modulus of the ionic crosslinked EPDM-g-MA is reported in Figure 4. As expected, the higher the KOH concentration, the higher the storage modulus. In fact, we can observe that at low frequencies, the storage modulus moves from 2 × 10^3^ Pa without KOH up to 1 × 10^5^ Pa for a ND of 75%. Generally speaking, a strong increase in the elasticity behavior can be observed. According to the model proposed by Eisenberg [13,31], the KOH is reacting with the maleic anhydride, and the ion pair tends to aggregate (inter- or intra-chain interaction), forming a physical network (Figure 3). This physical structuration limits the polymers chains’ mobility in comparison with the bulk neat polymer. The ionic-rich domains act as crosslinks and points of reinforcement for the elastic properties. These observations are clearly in agreement with the results reported by Li et al. [30] dealing with thermoreversible K ionomers based on butyl rubber. Based on the reaction between the maleic anhydride and the KOH, they demonstrated that the reprocessability of their system is observable for a degree of neutralization up to 70% (75% in our case) and that the ionic domains are microphase separated as evidenced by X-ray diffraction (XRD) analysis.

**Figure 5:**
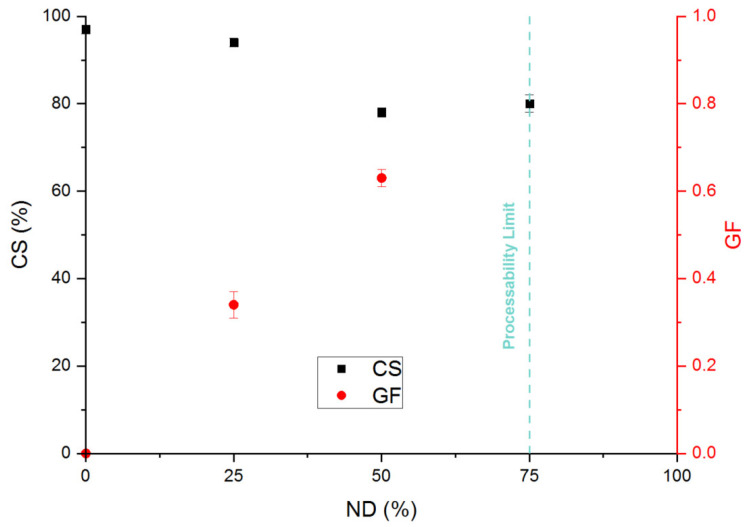
Variation of the compression set (■) and the gel fraction (●) for the EPDM-g-MA crosslinked with different KOH concentration (ND). No DCP has been added in these formulations.

Once again, the higher the KOH concentration, the higher the gel fraction and so the crosslinking density (Figure 5). We reached a maximum of 63% for a ND of 50%. Qualitatively, one can then expect a more pronounced dissipative effect compared with the chemical network and consequently poor recovery elasticity properties. Actually, the limit of the compression set that has been reached is 80% (Figure 5). Van der Mee et al. [17,18] show some similar results on EPDM-g-MA neutralized with KAc. Indeed, a compression set of 100% has been measured at 100 °C for the EPMD-g-MA alone. For an ND at 100, the compressions set at 39%. The same tendencies are observed: the higher the metallic salt concentration, the lower the compression set values. They also observed that the samples with an ND-100 are not completely homogenous. This is similar to what was observed in this study, namely that the sample at ND-100 is not processable. Furthermore, the optimum KOH concentration corresponds to a neutralization degree of 50%. Over this concentration, the compression set remains constant and around 80%. This result means that an ionic network does not lead to relevant elastic recovery properties, even if the storage modulus values correspond to a high elastic behavior. We have to be aware that the complex shear modulus is measured under linear deformation (*γ* < 0.05), whereas the compression set is measured under nonlinear condition (*ε*_0_ = 0.25). Consequently, under the nonlinear conditions of the compression set, the ionic network can be reorganized and rearranged (due to its ionic nature) losing a part of recoverable elasticity. However, the positive effect associated to this dynamic ionic system is that all the samples can make a film under large deformation. The ionic bonds allow chain mobility, which explains the capacity to be recycled.

Finally, whatever the crosslinking route (ionic or radical networks), it is not possible to obtain significant compression set properties while maintaining the processing property of the EPDM samples. In fact, a recovery elasticity target could be a compression set lower than 50%; unfortunately, the results show that it is not possible to go lower than 80% while being processable, in our experimental conditions.

### 3.3. Combination of Covalent and Ionic Networks

#### 3.3.1. KOH-Based Systems

**Figure 6:**
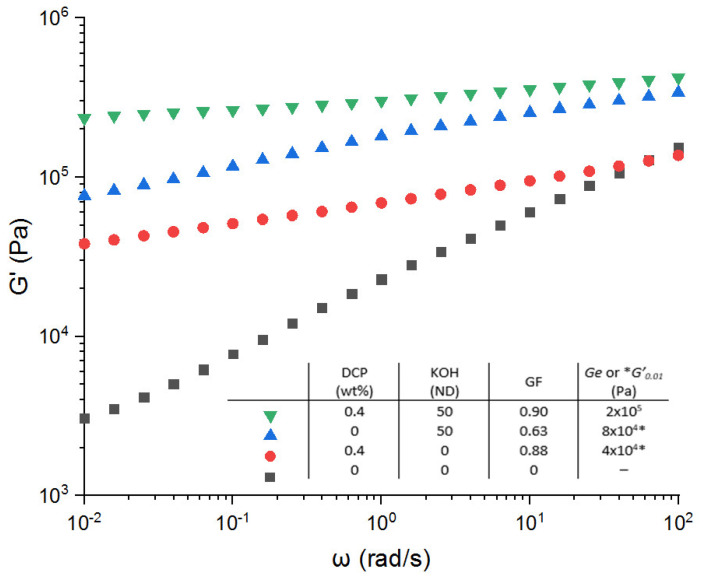
Variation of the storage modulus versus angular frequency. Comparison of the network types and influence of the combination of both ionic and covalent networks. * is *G*′_0_._01_ reported instead of *Ge*.

According to the works of Mora-Barrantes et al. [21], an improvement of the elasticity recovery from the combination of both covalent and ionic networks can be expected. Our main objective is to maintain the processable properties of the crosslinked samples, so we fixed the DCP concentration at 0.4 wt.%. As a result, Figure 6 shows the variation of the storage modulus for the covalent network (0.4 wt.% DCP, from Figure 1), the KOH-based ionic network (ND = 50 from Figure 4), and the combination of both networks. The cooperative effect of both networks can be pointed out. The storage modulus is higher with both crosslinking systems in comparison with the EPDM-g-MA alone or with only one crosslinking agent. For example, a crosslinking density of 67 mole/m^3^ is measured with the double crosslinking (*Ge* = 2 × 10^5^ Pa). This is much higher compared with the crosslink density of the respective network (*ν* = 11 mol/m^3^, *Ge* = 4 × 10^4^ Pa for the covalent system and *ν* = 25 mol/m^3^, *Ge* = 8 × 10^4^ for the KOH ionic system).

**Figure 7:**
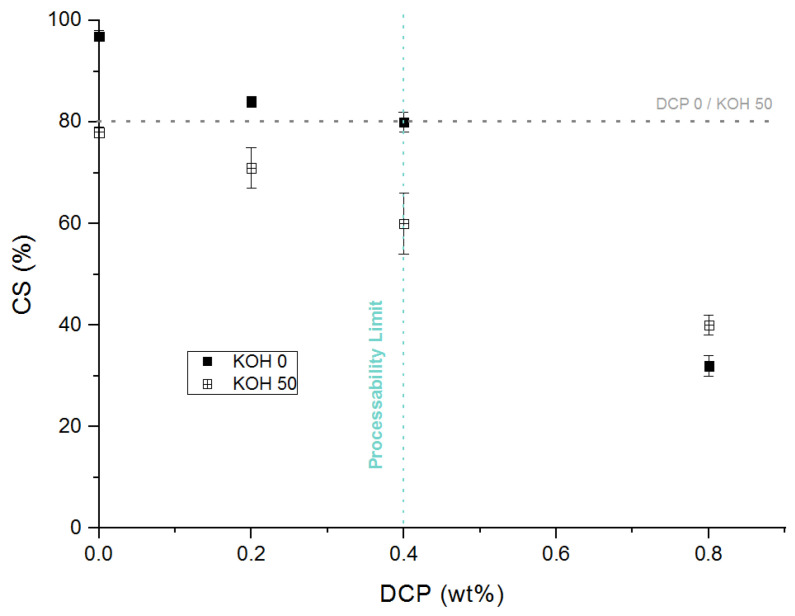
Variation of the compression set versus DPC concentration. Influence of the combination of both ionic and covalent networks.

Furthermore, the elastic recovery properties are considerably improved, as shown in Figure 7. For example, for 0.4 wt.% of DCP, the compression set decreases from 80% to 60% with the combination of both networks. Furthermore, this cooperative effect is observed at the different concentrations of DCP.

One explanation of such behavior has been addressed by Mora-Barrantes et al. [21] who studied the impact of both DCP and MgO on XNBR crosslinking. They explained that the covalent bonds reduce the mobility of the polymers chains and thus modify the aggregation of the ionic domain. As a consequence, the mixed systems are formed by more ionic crosslinked domains but they are smaller in size than for the pure ionic systems. This is demonstrated by the decrease in the aggregation number for the mixed systems [15] for a pure ionic system—4 phr of MgO—versus 6 for the mixed sample—4 phr of MgO and 2 phr of DCP). The changes in mechanical properties depend of various factors, but one of the most important is the network structure. The synergic effect between both types of crosslinking allowed an evolution of the network structure, which is beneficial for the compression set properties. Finally, with the twin crosslinking system, we managed to decrease until 60% of the compression set (versus 80% with only the covalent network or only the ionic network).

#### 3.3.2. Impact of the Nature of Ions

From these cooperative effects of combined networks, it is of importance to study the influence of the counterion nature and consequently the most appropriate one in terms of elasticity recovery. The effects of the combination of both crosslinking systems on the gel fraction, storage modulus, and compression set were then examined.

**Figure 8:**
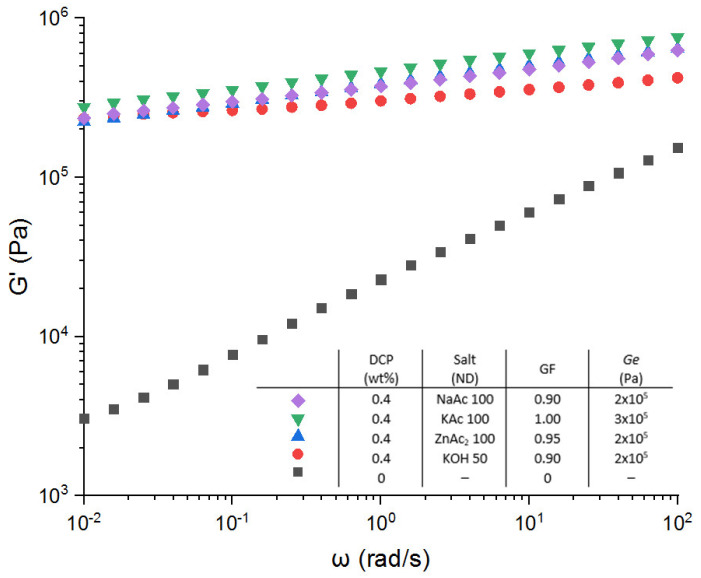
Storage modulus versus angular frequency for different salts in the case of the combination of covalent and ionic networks.

**Figure 9:**
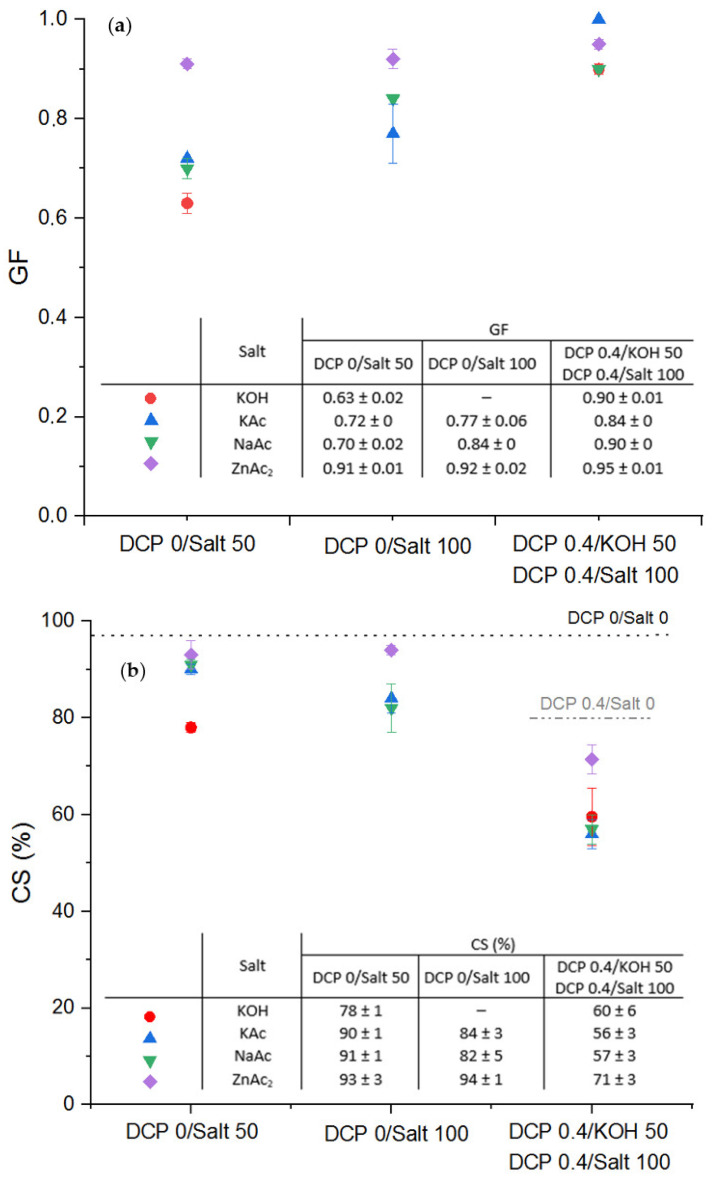
Variation of the gel fraction (**a**) and the compression set (**b**) of EPDM-g-MA crosslinked with different ions (KOH, KAc, NaAc, and ZnAc_2_) and with the combination of both ionic and covalent networks.

First of all, Figure 8 shows the storage modulus of the EPDM-g-MA with 0.4 wt.% of DCP and neutralized at 100% with the different salts (except KOH, which is neutralized at 50%). For all ions used, no real difference can be observed between the four salts (KOH, KAc, ZnAc_2_ and NaAc). The gel fraction (Figure 9a) in the range between 90 and 100% which clearly evidenced the crucial contribution of the covalent crosslinking. It must be pointed out that all samples can be processed.

Furthermore, it can be noticed that the ZnAc_2_ allowed obtaining the highest values of gel fraction (more than 90% against 63% for the KOH with a DN of 50%) of the sample gel fraction. Surprisingly, these differences observed on the GF are not observable on the storage modulus. These results highlight that a divalent cation (Zn^2+^) is more favorable to reach high gel fraction than the use of monovalent ions (K^+^ and Na^+^). This can be actually related to the specific coordination behavior of the Zn^2+^ cation with the dicarboxylate moieties of the EPDM-g-MA. This aspect was evidenced for example by Grady et al. [32], who depicted that the Zn^2+^ cation would preferentially coordinate with two carboxylate groups from two distinct maleic anhydride species, which can thus lead to a high gel fraction. These results are consistent with the observations reported by Stevens et al. [33] and Bragrodia et al. [34]. These former authors also described the bivalent Zn^2+^ cation as the more “covalent” character of the zinc. For the monovalent cation, the difference between the K^+^ and Na^+^ action is less pronounced in particular for the DN = 50, as for example, the size of the cation is in the same range (102 and 140 pm for respectively the sodium and potassium cations). Similarly, Wang [25] demonstrated a better shape memory effect of polyacrylate crosslinked by zinc salt. The nature of the counterion has to be considered. Likewise, the acetate counterion is favorable to increasing the gel fraction relative to the hydroxide when there is no combination with covalent crosslinking. This is coherent with the results shown by Wang et al. [25]. The acetate is the counterion that shows the best enhancement effect on tensile properties of Zn ionomers. The large anion group contributes to the dissociation of zinc salts, which can explain the best results observed with the acetate. Finally, we can observe that these differences are leveled when the combination covalent/ionic crosslinking reactions are used.

However, the expected correlation between gel fraction and compression set was not observed (Figure 9a,b): namely, the higher the crosslinking rate, the better the compression set. In fact, ZnAc_2_ has the best crosslinking rates, yet it has the poorest compression set. The same phenomenon was related by Van der Mee et al. [17], who compared the ionic crosslinking of an EPDM-g-MA with respectively the ZnAc_2_ and KAc. Actually, they showed that the tensile properties and elasticity in particular of the compression set are better for the K ionomers than for the Zn ionomers due to the relatively weak microphase separation for the latter ionomers. This could also be explained by the characters “hard” and “soft” of the acids defined by Bagrodia et al. [34]. Indeed, Zn^2+^ is at the limit between hard and soft acids, and K^+^ and Na^+^ are very hard acids. The carboxylate sites should associate more strongly with the potassium/sodium acetate than with the zinc acetate. It also has been demonstrated by Stevens et al. [33] that monovalent and divalent ions do not prefer the same number of ligands. Zn^2+^ prefer larger clusters (*n* = 3), while K^+^ and Na^+^ prefer smaller clusters with *n* = 2 ligands. So, the samples have different network structures, which can explain the differences observed in the elastic recovery properties compared with gel fractions. The cooperative effect between both types of crosslinking observed previously for the KOH/DCP-based system is confirmed here for all the other salt-based systems. The evolution of the network structure is beneficial for the compression set properties. However, according to the previous observations, the compression set for the system elaborated with the ZnAc_2_ remains higher even with the contribution of the DCP covalent crosslinking (minimum of 70%). Finally, with the double crosslinking system, we managed to decrease until 55% of the compression set for the monovalent cation-based system (versus 80% with only the covalent network or only the ionic network). This confirms the major influence of the nature of the cation and its ability to enhance a micro-phase separation.

All these samples are processable, so a compromise is obtained at 55% of the compression set.

#### 3.3.3. Water Aging

In terms of real and industrial applications, the question concerning the water resistance of these ionic bonds and their dissociation over time and under high temperature (80 °C) conditions is fundamental.

**Figure 10:**
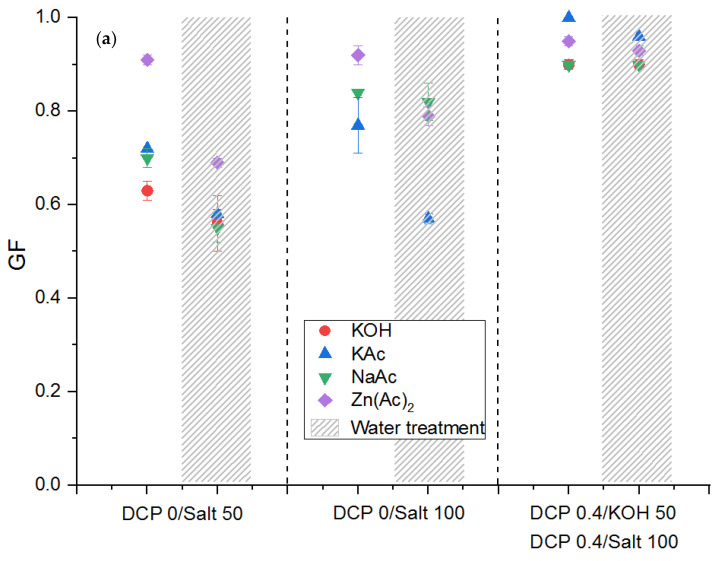

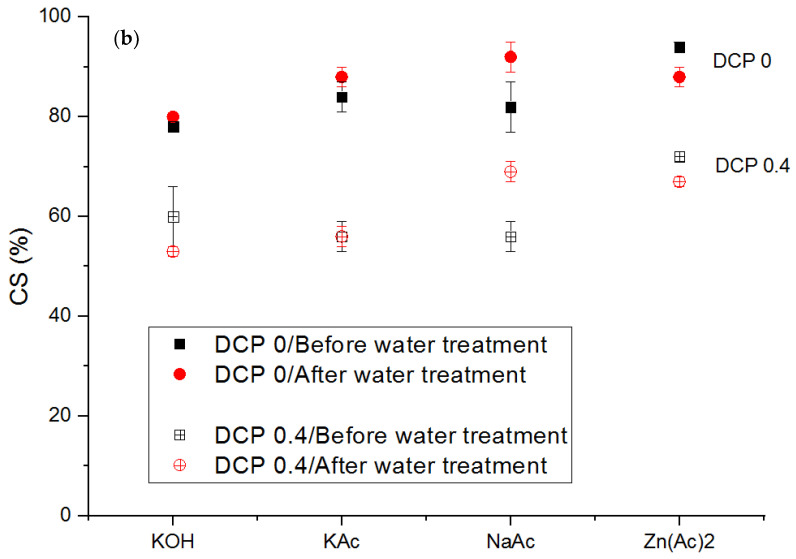
Variation of the gel fraction (**a**) and the compression set (**b**) of EPDM-g-MA crosslinked with different ions (KOH, KAc, NaAc, and ZnAc_2_) and with the combination of both ionic and covalent networks. Influence of the water treatment.

Thus, the influence of the water (48 h of immersion in water at 80 °C), depending on the ions used for the neutralization, is shown in Figure 10. We can notice that when the samples are ionically crosslinked, a decrease in the gel fraction is observed after the water treatment for all the metallic salts used (Figure 10a). This observation can be related to the well-known water solubility of these salts. Classically, at room temperature, the water solubility can be classified as from the less soluble to the most soluble: NaAc < ZnAc_2_ < KOH < KAc. To illustrate, for the KAc-based ionic system with a DN = 100, the gel fraction decreases drastically from almost 80% to less than 60%. In the same conditions, the gel fraction is almost not changed for the use of NaAc. However, in the presence of both networks, this decrease is not noticeable. Actually, as discussed by Mora et al. [21,35], the dual crosslinking action leads to smaller ionic domains, which can be less accessible to water molecules. In that case, the gel fraction remains almost unchanged in our water action conditions.

It can be noticed for all the salts used, except for NaAc, that the water treatment in our experimental conditions has no influence on the compression set (Figure 10b). On the contrary, with the NaAc, a degradation of the mechanical properties after water treatment is observed. The compression set increases by almost 20% after the water treatment with or without the covalent crosslinking with peroxide, even if this salt is the less water soluble of our series. Van deer Mee et al. [18] discussed specifically this water action in the case of EPDM-g-MA crosslinked by respectively ZnAc_2_ and KAc. They described that the water absorption can induce two competing effects: either opening residual maleic anhydride to yield two carboxylic acid groups, giving the capability to form hydrogen bonds or the promotion of the acid–cation exchange relaxation mechanism as described by Vanhoorne and Register [36]. In this publication, the former authors in particular demonstrated that unneutralized acid groups substantially reduce the viscosity of Na ethylene-methacrylic acid (EMAA)-based ionomers through this acid–cation exchange mechanism or through “plasticization” of the ionic aggregates but did not affect the viscosity of the similar Zn ionomers. Some other studies [37] in the literature present the same influence of water on ionomeric ethylene with different salts and are consistent with our experiments. Indeed, the EMAA neutralized with zinc does not absorb water, which explains the constancy of the results on stiffness and the small water contents of the samples aged in water (0.22% for 90 days at 100% of relative humidity). On the other hand, the EMAA neutralized with sodium absorbs more water, which will disturb the aggregates and thus the properties (decrease in stiffness in the presence of water and 3.80% of water for 90 days at 100% of relative humidity). For the potassium, the effect of water is not very clear, because the samples tend to absorb water similarly to sodium, but we do not see any changes.

## 4. Conclusions

The objective of this work was to improve the mechanical properties of an EPDM-g-MA (more precisely, the compression set) while keeping the processability of the samples. In that frame, the influence of a covalent and an ionic-reversible network has been studied. Samples crosslinked with DCP have the expected recovery elasticity but to the detriment of the processability. On the other hand, the ionic networks can be processed but with limited recovery plasticity properties (CS = 80%).

Combining both networks, an improvement of the recovery elasticity (lower compression set values) is observed while maintaining the elasticity recovery properties. Actually, there is a compromise between the processability and relevant mechanical properties (CS around 55%). This brings forth a cooperative effect between the two types of crosslinking. The influence of the cations (K^+^, Na^+^, and Zn^2+^) is compared, and the monovalent ions seem to be the best candidates in order to improve the elastic properties. The water aging was also studied, and it was observed that potassium acetate combines both good elastic recovery properties and water resistance properties. Finally, a compression set of 55% is obtained while maintaining processing processability without any negative effect of water treatment.

## Figures and Tables

**Table 1:** Characteristics of the EPDM-g-MA.

*T*_g_^(a)^	Maleic Anhydride ^(a)^	Propylene Ratio ^(b)^	Ethylene Ratio ^(b)^	Diene Ratio ^(b)^	Mn ^(c)^	Mw ^(c)^
(°C)	(wt.%)	(%)	(%)	(%)	(g/mol)	(g/mol)
−46	1	31	66.9	2.1	72,000	212,000

^(a)^ Supplier data, ^(b)^ Determined by ^1^H NMR, ^(c)^ Determined by SEC in our laboratory.

**Table 2:** Characteristics of the different blends. ND is the neutralization degree of the maleic anhydride on the polymer. GF is the gel fraction, *Ge* is the equilibrium modulus, and * *G*′_0_._01_ is the storage modulus *G*′ measured at the lowest frequancy, i.e., *ω* = 0.01 rad/s.

Name	Metallic Salt	ND	DCP (wt.%)	GF	*Ge*/*G*′_0.01_ (Pa)
DCP 0/Salt 0	-	-	-	0	-
DCP 0.2/Salt 0	-	-	0.2	0.57	2.16 × 10^4^ *
DCP 0.4/Salt 0			0.4	0.88	3.82 × 10^4^ *
DCP 0.8/Salt 0			0.8	0.90	1.25 × 10^5^
DCP 1.5/Salt 0			1.5	0.95	1.79 × 10^5^
DCP 0/KOH 25	KOH	25	-	0.34	3.43 × 10^4^ *
DCP 0/KOH 50		50		0.63	7.64 × 10^4^ *
DCP 0/KOH 75		75		- **	1.05 × 10^5^ *
DCP 0/KOH 100		100		Powder	Powder
DCP 0.2/KOH 50	KOH	50	0.2	0.88	1.68 × 10^5^
DCP 0.4/KOH 50			0.4	0.90	2.34 × 10^5^
DCP 0.8/KOH 50			0.8	0.92	2.63 × 10^5^
DCP 0/ZnAc_2_ 50	ZnAc_2_	50	-	0.91	-
DCP 0/ZnAc_2_ 100		100	-	0.92	5.35 × 10^4^ *
DCP 0.4/ZnAc_2_ 100		100	0.4	0.95	2.23 × 10^5^
DCP 0/KAc 50	KAc	50	-	0.72	4.78 × 10^4^ *
DCP 0/KAc 100		100	-	0.77	6.25 × 10^4^ *
DCP 0.4/KAc 100		100	0.4	1.00	2.76 × 10^5^
DCP 0/NaAc 50	NaAc	50	-	0.70	2.27 × 10^4^ *
DCP 0/NaAc 100		100	-	0.84	6.76 × 10^4^ *
DCP 0.4/NaAc 100		100	0.4	0.90	2.35 × 10^5^

* *G*′_0_._01_ is reported, ** The gel fraction cannot be measured.
